# LncRNA PCGEM1 contributes to malignant behaviors of glioma by regulating miR-539-5p/CDK6 axis

**DOI:** 10.18632/aging.202476

**Published:** 2021-02-11

**Authors:** Shu-Li Liu, Mao-Hua Chen, Xiao-Bo Wang, Rong-Kai You, Xue-Wei An, Qiu Zhao, Ren-Shu Wang

**Affiliations:** 1 Department of Intensive Care Unit, Wenzhou Central Hospital, Wenzhou Medical University, Wenzhou 325000, China; 2 Neurosurger Department, Wenzhou Central Hospital, Wenzhou Medical University, Wenzhou 325000, China; 3 Wenzhou Seventh People’s Hospital, Wenzhou 325000, China; 4 Emergency Department, The First Affiliated Hospital of Wenzhou Medical University, Wenzhou 325000, China

**Keywords:** glioma, PCGEM1, miR-539-5p, CDK6, progression

## Abstract

Background: Glioma, one of the most prevalent and aggressive cancers, is regulated by long noncoding RNAs (lncRNAs). This study aims to research the functional mechanism of lncRNA PCGEM1 involved in glioma progression.

Methods: Expression levels of PCGEM1, miR-539-5p and CDK6 were analyzed by qRT-PCR in NHA, U251, U87, and LN229 cells or glioma tissues. shRNAs were used to knock down PCGEM1 in U251 and LN229 cells. Kaplan-Meier curve and log rank test were utilized to examine survival rate. CCK8 (Cell Counting Kit-8) assay, colony formation assay and EdU staining were conducted to detect cell proliferation. Transwell assay was performed to evaluate cell migration and invasion. Luciferase reporter assay was conducted to assess RNA interaction between PCGEM1 and miR-539-5p. Nude mice were used for tumor xenograft assay.

Results: LncRNA PCGEM1 was upregulated in glioma tissues and tumor cell lines. PCGEM1 upregulation predicted unsatisfactory prognosis. PCGEM1 knockdown inhibited proliferation, colony formation, migration and invasion. PCGEM1 knockdown delayed tumor growth *in vivo*. PCGEM1 played as a competing endogenous RNA (ceRNA) for miR-539-5p to promote CDK6 expression. MiR-539-5p mimics repressed glioma progression while CDK6 overexpression reversed the roles of PCGEM1 knockdown.

Conclusion: PCGEM1 knockdown suppressed glioma progression through sponging miR-539-5p and regulating CDK6 expression, implying PCGEM1 as a potential therapeutic target.

## INTRODUCTION

Glioma is one of the most prevalent and aggressive tumor cancers and results in a great number of cancer-associated deaths worldwide [1]. Glioma accounts for about 80% of all tumors in the central nervous system [2]. The overall survival rate of glioma patients still remains very low [3]. According to the pathologic growth and diffusion velocity features, glioma could be graded into I–IV [4]. However, most patients are diagnosed at advanced stages and metastasis occurs, which makes treatment more difficult. Hence, it is urgent to understand the molecular mechanism underlying glioma progression and develop novel therapeutic targets.

Long noncoding RNAs (lncRNAs) are characterized by 200 nucleotides in length and limited protein-coding potential [5]. Growing studies suggest that lncRNAs regulate multiple biological roles, such as cell growth, metastasis and apoptosis [6]. LncRNA may work as the functional molecule and serve as a completing endogenous RNA (ceRNA) for miRNAs [7]. They could restrain miRNA activity to regulate mRNA level [7]. Dysregulation of lncRNA is observed to affect tumorigenesis. For example, LINC01638 is upregulated in liver cancer and enhances tumor growth through regulating glucose uptake [8]. LncRNA UCA1 interacts with miR-28-5p to regulate HOXB3 expression and promotes colon cancer growth and invasion [9]. In addition, lncRNA UCA1 regulates metastasis of tongue tumor cells by sponging miR-124 [10]. In view of the pivotal functions of lncRNAs in cancer, it is important to investigate how lncRNAs participate in glioma progression.

PCGEM1 is discovered to promote prostate cancer progression [11]. Recent studies also revealed its oncogenic functions in gastric cancer and endometrial carcinoma [12, 13]. Our study for the first time explored the role of PCGEM1 in glioma cells. We demonstrated that PCGEM1 level was increased in glioma tissues and its knockdown suppressed glioma proliferation, migration and invasion *in vitro*. We showed that PCGEM1 promoted CDK6 expression through inhibiting miR-539-5p. Summarily, our findings suggest that PCGEM1 plays oncogenic functions in glioma cells through modulating miR-539-5p/CDK6 pathway.

## RESULTS

### PCGEM1 expression in glioma cells

**Figure 1:**
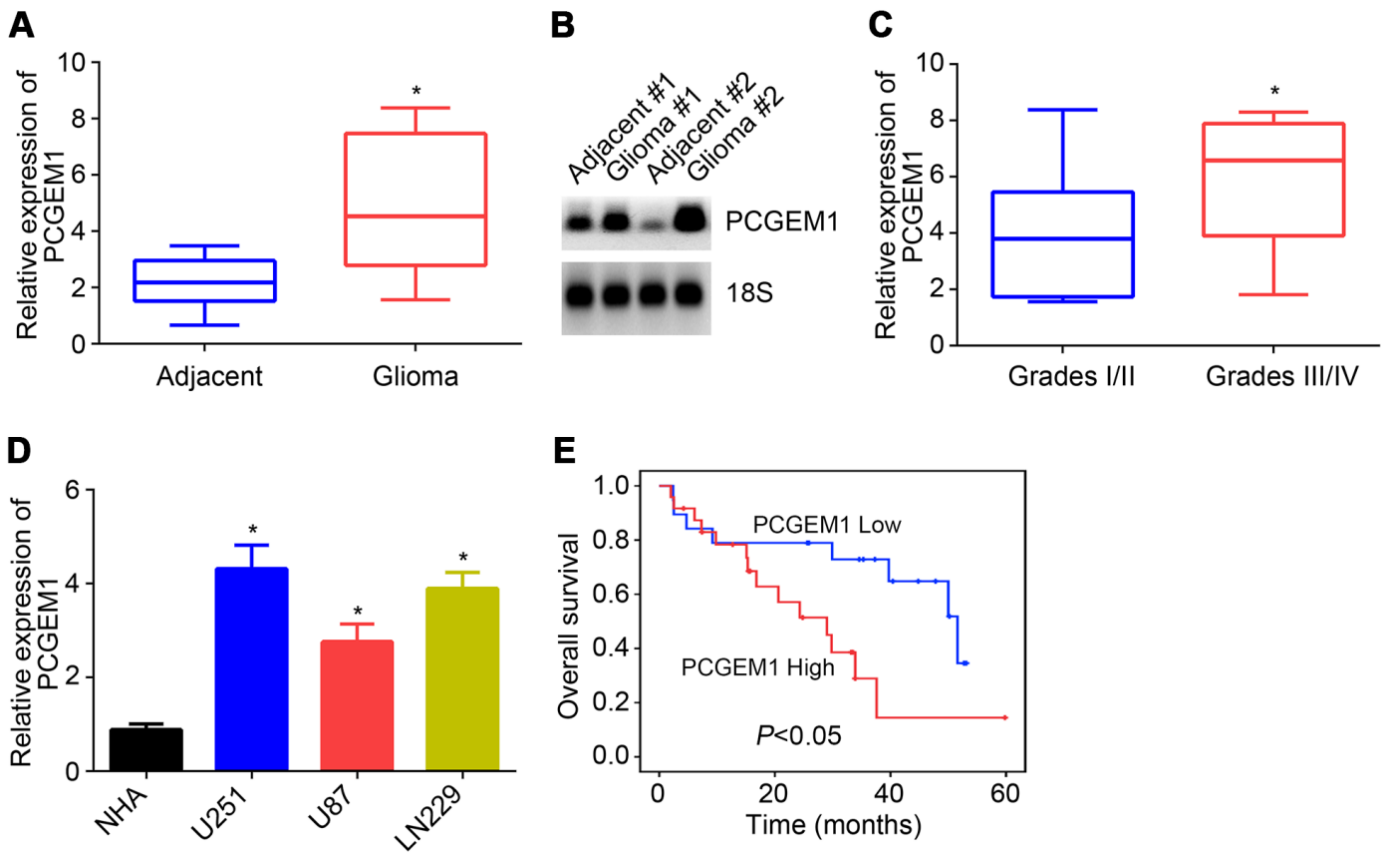
**PCGEM1 expression in glioma.** (**A**) PCGEM1 expression in glioma tissues and adjacent normal tissues. (**B**) Northern blot was performed to analyze PCGEM1 expression in glioma tissues. (**C**) Relative expression of PCGEM1 in glioma tissues with different grades. (**D**) PCGEM1 expression in glioma cell lines was analyzed. (**E**) Survival rate was examined based on PCGEM1 expression. *P<0.05.

The expression of PCGEM1 in 43 glioma tissues and their corresponding adjacent normal tissues was analyzed through qRT-PCR. Results showed that PCGEM1 level was increased in glioma tissues (Figure 1A), which was confirmed by Northern blot analysis (Figure 1B). These glioma tissues were divided into two subgroups based on WHO grades. qRT-PCR indicated that PCGEM1 expression was higher in glioma tissues with graded III/IV (Figure 1C). qRT-PCR found that PCGEM1 levels were raised in glioma cell lines compared to NHA cells (Figure 1D). Finally, the survival rate of patients with glioma observed in the clinic was analyzed based on PCGEM1 expression. Results showed that PCGEM1 high expression was associated with poor prognosis (Figure 1E).

### Effects of PCGEM1 knockdown on glioma cells

**Figure 2:**
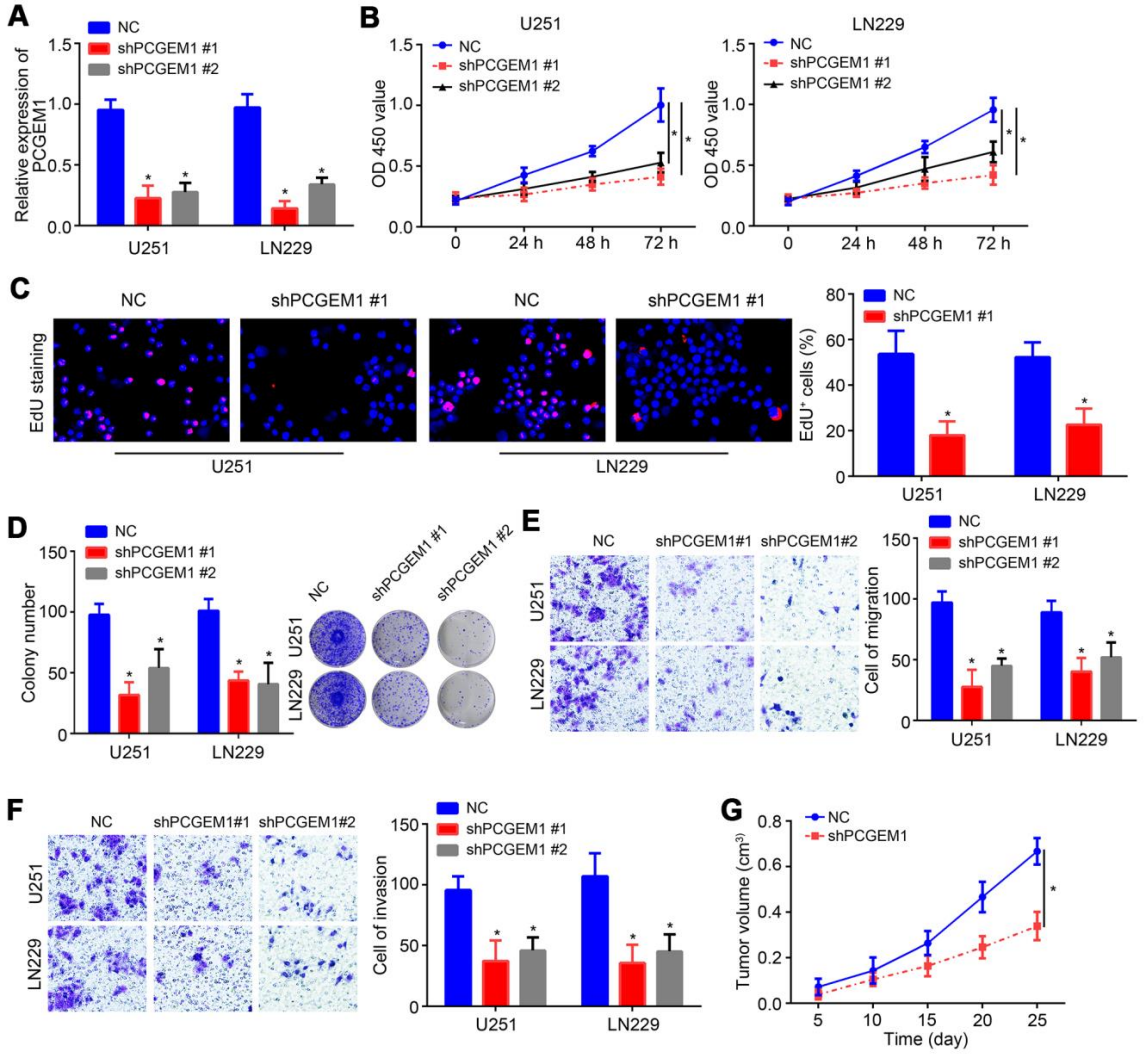
**Effects of PCGEM1 knockdown on glioma cells.** (**A**) PCGEM1 expression was knocked down using shRNA targeting PCGEM1. (**B**) CCK8 assay for proliferation assessment. (**C**) EdU staining for assessing proliferation. (**D**) Colony formation assay was performed to evaluate proliferation. (**E**, **F**) Transwell assay for migration and invasion. (**G**) Animal xenograft experiment assay was performed and tumor volumes were measured at indicated time points. *P<0.05.

To investigate the role of PCGEM1, shRNA targeting PCGEM1 was utilized and transfected into U251 and LN229 cells. PCGEM1 expression was successfully knocked down by shPCGEM1 (Figure 2A). Results from CCK8 assay showed that PCGEM1 knockdown delayed glioma cell growth (Figure 2B). The EdU positive cells were fewer in shPCGEM1 group than that in NC group (Figure 2C). Colony formation assay also confirmed that PCGEM1 knockdown inhibited glioma cell proliferation (Figure 2D). Transwell assay showed that PCGEM1 knockdown suppressed the cell numbers of migration and invasion (Figure 2E, 2F). Moreover, animal xenograft experiment was performed and PCGEM1 knockdown delayed tumor growth *in vivo* (Figure 2G).

### PCGEM1 was the ceRNA for miR-539-5p

**Figure 3:**
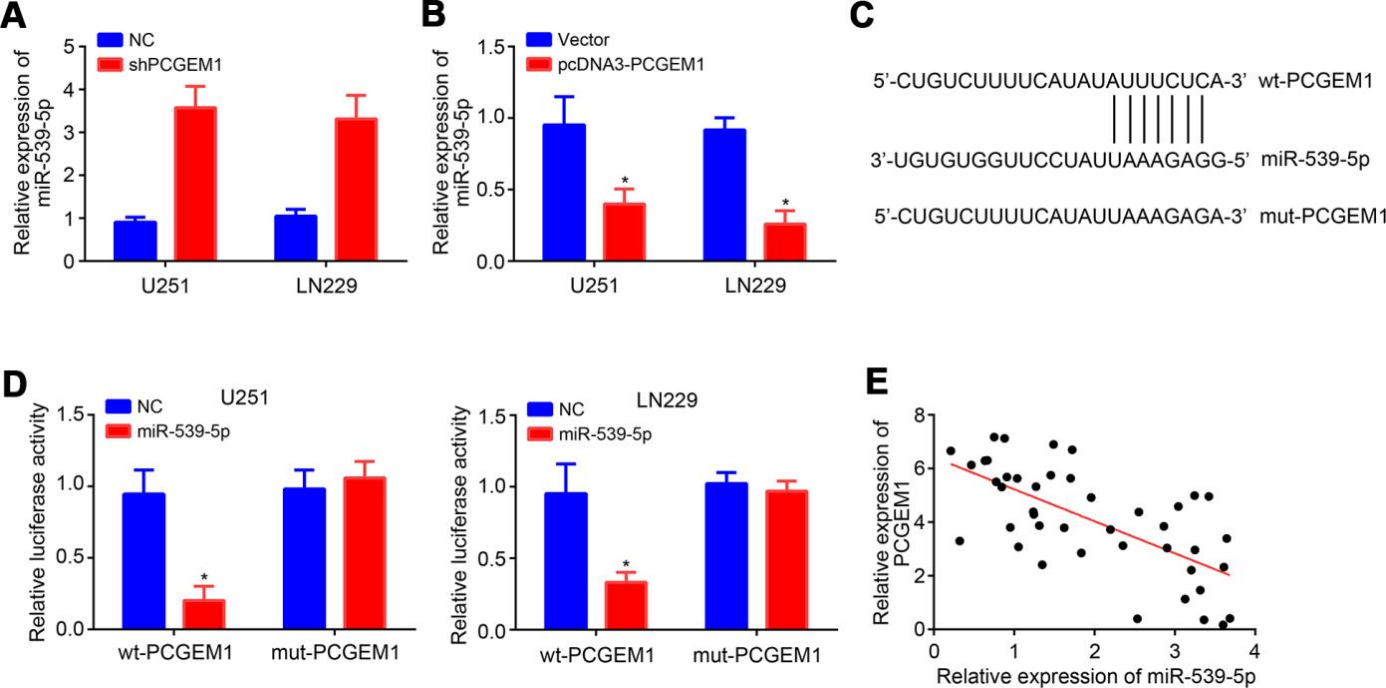
**PCGEM1 was the ceRNA for miR-539-5p.** (**A**, **B**) Relative expression of miR-539-5p after PCGEM1knockdown or overexpression. (**C**) Predicted binding site in PCGEM1 with miR-539-5p through miRDB. (**D**) Luciferase reporter assay was performed. (**E**) Correlation between PCGEM1 and miR-539-5p expressions in glioma tissues (r=-0.6704; P<0.001; R square=0.4494). *P<0.05.

We further analyzed the potential miRNA target of PCGEM1 through miRDB tool. We identified miR-539-5p as the most potential candidate because it ranked top with the highest score. qRT-PCR analyses showed that PCGEM1 knockdown led to increased expression of miR-539-5p and vice versa (Figure 3A, 3B). Then, the wild-type (wt) and mutant (mut) luciferase reporter vectors were constructed (Figure 3C). Results showed that miR-539-5p mimics suppressed the activity of wt-PCGEM1 reporter in U251 and LN229 cells (Figure 3D), suggesting that PCGEM1 directly interacts with miR-539-5p. qRT-PCR results also indicated that miR-539-5p expression was reversely correlated with PCGEM1 in glioma tissues (Figure 3E).

### MiR-539-5p targeted CDK6

**Figure 4:**
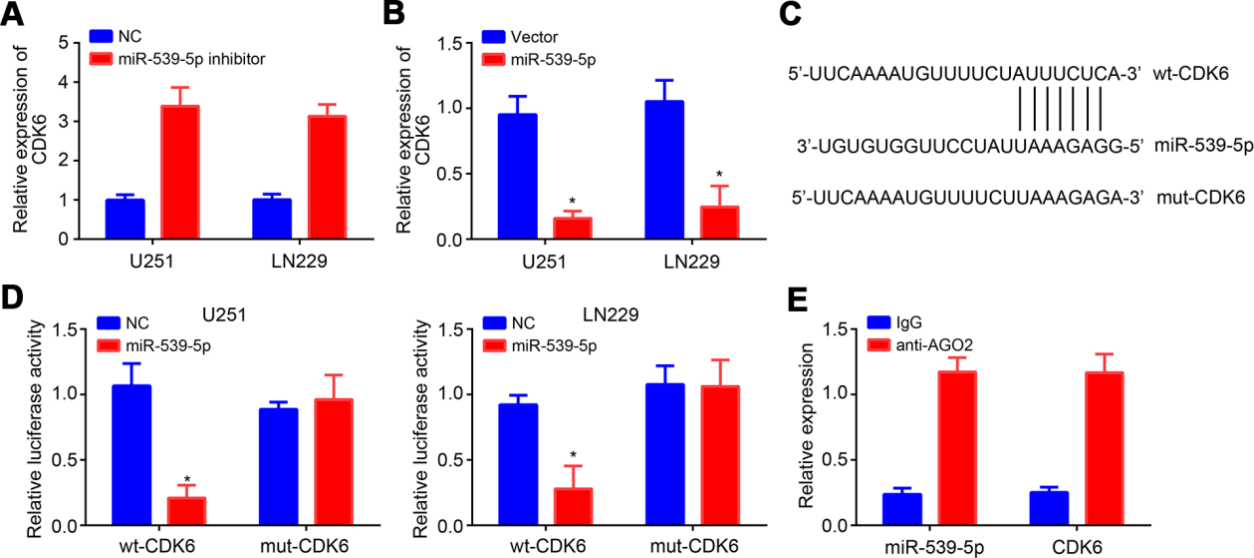
**MiR-539-5p targeted CDK6.** (**A**, **B**) Relative expression of CDK6 after transfection with miR-539-5p inhibitors or mimics. (**C**) Predicted binding site in CDK6 with miR-539-5p through TargetScan. (**D**, **E**) Luciferase reporter assay and RIP assay were conducted to validate the interaction between miR-539-5p and CDK6. *P<0.05.

Afterwards, TargetScan tool was used to predict the target mRNA of miR-539-5p. CDK6 ranked top with the highest score. MiR-539-5p inhibition resulted in raised expression of CDK6 and vice versa (Figure 4A, 4B). Similarly, wt and mut-CDK6 luciferase reporter vectors were generated (Figure 4C). Luciferase reporter assay demonstrated the direct interaction between miR-539-5p and CDK6 (Figure 4D), which was further validated by RIP assay (Figure 4E).

### PCGEM1 promoted glioma progression through miR-539-5p/CDK6 pathway

**Figure 5:**
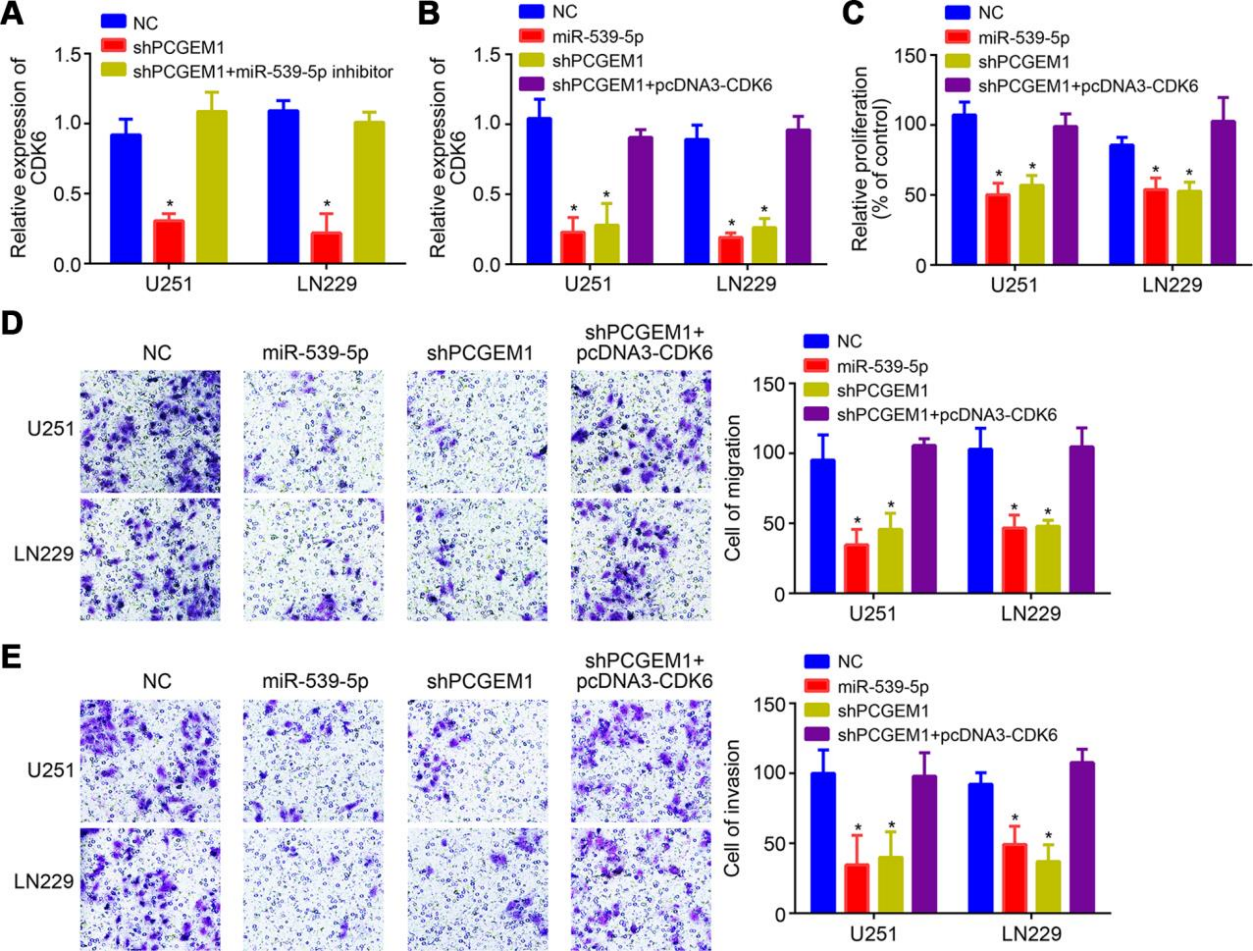
**PCGEM1 promoted glioma progression through miR-539-5p/CDK6 pathway.** (**A**, **B**) Relative expression of CDK6 after transfection of indicated vectors. (**C**) CCK8 assay for proliferation. (**D**, **E**) Transwell assay for analysis of cell migration and invasion. *P<0.05.

It was observed that PCGEM1 knockdown suppressed CDK6 expression while miR-539-5p inhibition rescued its expression (Figure 5A), indicating that PCGEM1 sponges miR-539-5p from CDK6 mRNA. Moreover, we noticed that miR-539-5p mimics or CDK6 silencing suppressed the proliferation, migration and invasion of glioma cells *in vitro* (Supplementary Figure 1A–1C). To explore whether PCGEM1 regulates glioma progression through miR-539-5p/CDK6 axis, CDK6 expression was rescued by transfection of pcDNA3-CDK6 vector (Figure 5B). Then CCK8 and transwell assays were conducted. Results showed that either PCGEM1 knockdown or miR-539-5p mimics suppressed glioma cell proliferation, migration and invasion while CDK6 overexpression reversed the effects of PCGEM1 knockdown (Figure 5C–5E). Therefore, PCGEM1 regulates miR-539-5p/CDK6 axis to promote glioma progression.

## DISCUSSION

Glioma is the most aggressive brain tumor and causes high mortality. However, its pathogenesis is still largely unknown. In this study, it was found that PCGEM1 was upregulated in glioma tissues and cell lines. And PCGEM1 upregulation predicted poor prognosis in glioma patients. PCGEM1 knockdown suppressed the proliferation, migration and invasion of glioma cells. PCGEM1 was found to sponge miR-539-5p to facilitate CDK6 expression. Our findings demonstrate that PCGEM1 functions as oncogenic roles through regulating miR-539-5p/CDK6 pathway.

Emerging research has found that lncRNAs are important molecules in regulating tumorigenesis by serving as oncogenes or cancer suppressors [14]. The roles of lncRNAs in glioma have also been discovered. For example, lncRNA OIP5-AS1 promotes glioma growth and metastasis through targeting miR-410/Wnt-7b axis [15]. LncRNA DLX6-AS1 upregulation enhances proliferation and invasion of glioma cells through sponging miR-197-5p to upregulate E2F1 expression [16]. LncRNA TP73-AS1 binds to miR-124 to relieve p53 and promote glioma progression [17]. The function of PCGEM1 is mainly researched in prostate cancer. Several reports support that PCGEM1 is an oncogene in prostate cancer [11, 18]. Recent findings reveal that PCGEM1 may also function in other cancers, such as gastric cancer and endometrial carcinoma [12, 13]. Nevertheless, PCGEM1 function in glioma is not researched. This study identified that PCGEM1 was upregulated in glioma tissues and cells. And PCGEM1 knockdown repressed the proliferation, migration and invasion of glioma cells, suggesting PCGEM1 is an oncogene.

Growing evidences have demonstrated that lncRNA-miRN-mRNA is an important regulatory manner in cancer [9, 17]. And miRNAs are crucial oncogenes or tumor suppressors in tumor. For example, lncRNA HULC interacts with miR-186 to promote HMGA expression, leading to liver cancer progression [19]. LncRNA LOXL1-AS1 modulates miR-541-3p/CCND1 pathway to promote prostate cancer growth and invasion [20]. In addition, lncRNA SNHG1 is the sponge for miR-145-5p to upregulate MTDH and enhance non-small cell lung cancer development [21]. In our study, we found that miR-539-5p was sponged by PCGEM1. Through luciferase reporter assay, we observed their direct interaction. PCGEM1 regulated miR-539-5p expression in glioma cells. MiR-539-5p has been reported to inhibit nasopharyngeal carcinoma development [22]. Our present study discovered the anti-cancer roles of miR-539-5p in glioma cells. We found that miR-539-5p is sponged by PCGEM1 and its upregulation suppressed glioma cell growth, migration and invasion.

Subsequently, CDK6 was proven as the target of miR-539-5p by bioinformatics, luciferase reporter assay and RIP assay. CDK6 is a classical oncogene and regulates cell-cycle progression in many types of cancers [23]. It has found that CDK6 positively regulates proliferation, migration and invasion of several cancer cells, such as esophageal cancer, gastric cancer and lung cancer [23–25]. Notably, previous work also highlighted the importance of CDK6 in glioma [26, 27]. Consistently, our results also showed that CDK6 overexpression promoted the proliferation, migration and invasion of glioma cells. Moreover, we revealed that CDK6 was regulated by PCGEM1 through inhibiting miR-539-5p in glioma cells.

## CONCLUSIONS

In sum, the present research discovered that PCGEM1 was an oncogene in glioma by modulating miR-539-5p/CDK6 pathway, suggesting that PCGEM1 may be a novel therapeutic target. Our study for the first time defined the role of PCGEM1in glioma and illustrated the regulatory relationship between PCGEM1 and miR-539-5p/CDK6 axis.

## MATERIALS AND METHODS

### Patients

43 glioma patients’ tissues (27 males and 16 females; mean age of 47.6±6.2 years) and adjacent normal tissues were collected from Wenzhou Central Hospital. None received other treatment before surgery. This study was approved by the ethics committee of Wenzhou Central Hospital. Experiments were performed in accordance with the Helsinki declaration. Written informed consent was obtained from very patient. Tissues were stored in liquid nitrogen until use.

### Cell culture and transfection

Glioma cell lines and normal human astrocyte cells (NHA) were obtained from the American Type Culture Collection (Manassas, VA, USA) and cultured using Dulbecco's modified Eagle's medium (DMEM) supplemented with 10% fetal bovine serum (FBS). Short hairpin RNA (shRNA) targeting PCGEM1, miR-539-5p mimics, miR-539-5p inhibitors and negative controls were obtained from Shanghai GenePharma Co., Ltd. Plasmids were transfected into cells using Lipofectamine® 2000 (Invitrogen; Thermo Fisher Scientific, Inc., CA, USA) following the manufacturer’s instructions.

### qRT-PCR

Total RNA was isolated using TRIzol and transcribed into complementary DNA using the RevertAid First Strand cDNA Synthesis Kit (Thermo Fisher Scientific, Inc., MA, USA). Then qPCR was carried out using the QuantiNova™ SYBR® Green PCR kit. GAPDH was the internal standard. The primer sequences were as follows: PCGEM1 (forward: 5’-CACGTGGAGGACTAAGGGTA-3’, reverse: 5’-TTGCAACAAGGGCATTTCAG-3’); miR-539-5p (5’-GGAGAAATTATCCTTGGTGTGT-3’); U6 (5’-GCTTCGGCAGCACATATACTAAAAT-3’); CDK6 (forward 5’-TGGAGACCTTCGAGCACC-3’ and reverse, 5’-CACTCCAGGCTCTGGAACTT-3’) and GAPDH (forward 5’-CATCACTGCCACCCAG-3’ and reverse 5’-ATGCCAGTGAGCTTCCC-3’).

### Cell proliferation assay

After transfection, cells were seeded into 96-well plates and cultured at indicated time. Then 10 μl of CCK-8 (Dojindo Molecular Technologies, Inc.) solution was added and incubated for 2 h. Cell proliferation was then evaluated by measuring the absorbance at 450 nm through a microplate reader (Bio-Rad Laboratories, Inc.).

### Colony formation assay

Transfected cells were seeded into 6-well plates and cultured for 14 days. Then cells were fixed with methanol and stained with 0.5% crystal violet. Colony number was counted finally.

### Transwell assay

Cell migration and invasion were assessed by transwell assay. For migration, transfected cells were placed into the upper chamber (BD Biosciences) with serum-free medium. For invasion, cells were added into the Matrigel®-pre-coated (Sigma-Aldrich; Merck KGaA) upper chamber. 600 μl medium containing 10% FBS was inoculated in the lower chamber. After cultured for 24 h, the migrated or invaded cells in the lower chamber was fixed with 4% paraformaldehyde and stained using 0.5% crystal violet. Cells were counted using an inverted light microscope.

### Tumor xenograft assay

6-week old female Nude mice were randomly divided into two groups (n=5 for each group) and inoculated subcutaneously on the right flank with 2×10^6^ U251 cells. Tumor volumes were measured every five days according to the formula: Tumor volume = (length×width^2^)/2. This experiment was approved by the Ethics Committee of Wenzhou Central Hospital.

### Luciferase reporter assay

The interaction between PCGEM1 and miR-539-5p was identified by miRDB. The interaction between CDK6 and miR-539-5p was predicted by TargetScan. The wild-type (WT) and mutant (Mut) sequences of PCGEM1 or CDK6 were cloned into o the pmirGlO Dual-luciferase miRNA Target Expression Vector (Promega, Madison, WI, USA). Then luciferase vector and miR-539-5p were co-transfected into the glioma cells. After 24 h, the luciferase activity was detected using a Dual-Luciferase Reporter Assay System (Promega).

### Statistical analysis

Results were expressed as the means ± standard deviation and analyzed through SPSS 22.0 (SPSS, Inc.). The significances of differences were analyzed through Student's t-test or one-way analysis of variance (ANOVA). Correlation between RNA levels was examined using Spearman's correlation analysis. P<0.05 was considered to indicate a statistically significant difference.

## Supplementary Material

Supplementary Figure 1
