# Supplementary material for: LncRNA PCGEM1 contributes to malignant behaviors of glioma by regulating miR-539-5p/CDK6 axis
Source: Aging (Albany NY). 2021 Feb 11;13(4):5475–84. doi: 10.18632/aging.202476 (PMC7950308; doi:10.18632/aging.202476)
Supplement: Supplementary Figure 1 [file aging-13-202476-s001.pdf]

SUPPLEMENTARY FIGURE

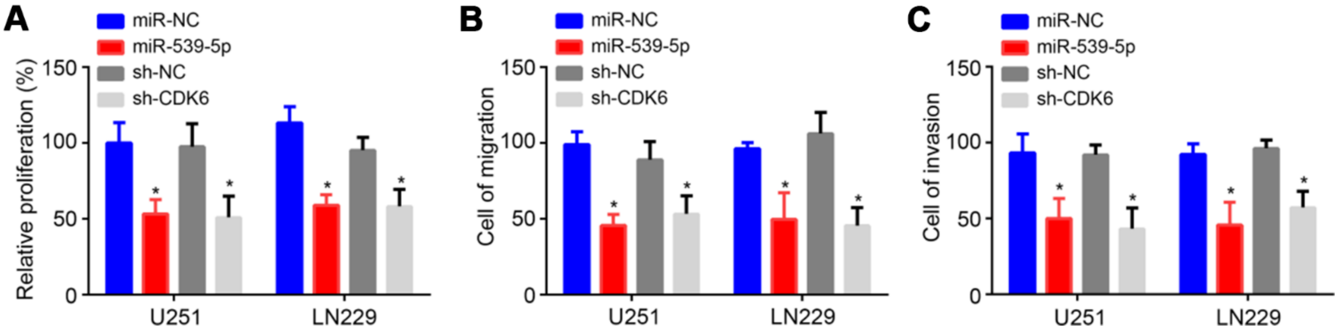

Supplementary Figure 1. Roles of miR-539-5p and CDK6 on glioma malignant behaviors. (**A**) CCK8 assay for proliferation assessment. (**B**, **C**) Transwell assay for migration and invasion. *P<0.05.
